# Molecular Characterization of Antimicrobial Peptide Genes of the Carpenter Ant *Camponotus floridanus*

**DOI:** 10.1371/journal.pone.0043036

**Published:** 2012-08-09

**Authors:** Carolin Ratzka, Frank Förster, Chunguang Liang, Maria Kupper, Thomas Dandekar, Heike Feldhaar, Roy Gross

**Affiliations:** 1 Department of Microbiology, Biocentre, University of Würzburg, Würzburg, Germany; 2 Department of Bioinformatics, Biocentre, University of Würzburg, Würzburg, Germany; 3 Department of Animal Ecology I, University of Bayreuth, Bayreuth, Germany; University Of Montana – Missoula, United State of America

## Abstract

The production of antimicrobial peptides (AMPs) is a major defense mechanism against pathogen infestation and of particular importance for insects relying exclusively on an innate immune system. Here, we report on the characterization of three AMPs from the carpenter ant *Camponotus floridanus*. Due to sequence similarities and amino acid composition these peptides can be classified into the cysteine-rich (e.g. defensin) and glycine-rich (e.g. hymenoptaecin) AMP groups, respectively. The gene and cDNA sequences of these AMPs were established and their expression was shown to be induced by microbial challenge. We characterized two different *defensin* genes. The *defensin-2* gene has a single intron, whereas the *defensin-1* gene has two introns. The deduced amino acid sequence of the *C. floridanus* defensins is very similar to other known ant defensins with the exception of a short C-terminal extension of defensin-1. The *hymenoptaecin* gene has a single intron and a very peculiar domain structure. The corresponding precursor protein consists of a signal- and a pro-sequence followed by a hymenoptaecin-like domain and six directly repeated hymenoptaecin domains. Each of the hymenoptaecin domains is flanked by an EAEP-spacer sequence and a RR-site known to be a proteolytic processing site. Thus, proteolytic processing of the multipeptide precursor may generate several mature AMPs leading to an amplification of the immune response. Bioinformatical analyses revealed the presence of *hymenoptaecin* genes with similar multipeptide precursor structure in genomes of other ant species suggesting an evolutionary conserved important role of this gene in ant immunity.

## Introduction

Insects have evolved multiple innate defense mechanisms to respond to microbial invasion [1], [2], [3], [4]. Early protective measure involves the “constitutive” immediate-acting defenses including phagocytes and reactive oxygen species. At later time points, an inducible immune response is mounted which mainly involves the production of antimicrobial peptides (AMPs) [5], [6]. It is believed that this late-acting humoral response is required to kill those bacteria that have survived the immediate host's constitutive defenses [5]. In 1981, the first AMPs were described from the cecropia moth [7]. In the past two decades a multitude of AMPs have been identified produced by many different organisms ranging from animals to plants. Most AMPs have a low molecular weight (<10 kDa), are membrane-active and display hydrophobic and/or cationic properties. Based on structural characteristics, insect AMPs can be divided into several groups, mainly α-helical peptides (e.g. cecropin), cysteine-rich peptides (e.g. defensin), proline-rich peptides (e.g. drosocin), and glycine-rich peptides (e.g. hymenoptaecin) [8].

Several AMPs from Hymenopteran species have been reported so far [9], [10], [11], [12], [13]. The few defensins known from Hymenopterans are short cationic peptides characterized by three stabilizing disulfide bridges. These peptides appear to act primarily against gram-positive bacteria by interference with acidic phospholipids of the cytoplasmic membrane and the formation of voltage-dependent channels [14], [15]. They are synthesized as an inactive precursor peptide with a signal- and a pro-sequence. Processing of the precursors leads to the active peptides. *Apis mellifera* has two structurally different *defensin* genes (*defensin-1* and *defensin-2*) [16]. *Defensin-1* is characterized by the presence of two introns and three exons, whereat the last exon encodes a short C-terminal extension known from bee defensins only. In contrast, *defensin-2* possesses a single intron [16]. Several *defensin-2* genes of a variety of ant species, including *Formica*, *Lasius* and *Myrmica* species, have been described previously [12]. The comparison and determination of codon substitution frequencies revealed positive selection in the mature region of the ant defensins, while the signal- and pro-regions of the AMPs appear to have evolved neutrally [12].

Hymenoptaecins are glycine-rich AMPs with activity against gram-negative and gram-positive bacteria and have been reported so far from Hymenopterans only [17]. The reported antibacterial effects of the *A. mellifera* hymenoptaecin suggest that its lethal consequences against *E. coli* are secondary to sequential permeabilization of the outer and inner membranes of these gram-negative bacteria [17]. The bee hymenoptaecins are endowed with a signal- and a pro-sequence which after processing give rise to the mature and active AMP [9], [10], [18]. In contrast, the two similar hymenoptaecins from the wasp *Nasonia vitripennis* encode multipeptide precursors with an AMP-like region at the position corresponding to the propeptide of bee hymenoptaecins [11], [19].

Here, we report the identification and molecular characterization of three AMP genes from the carpenter ant *Camponotus floridanus*. All ants are eusocial. Colonies of *C. floridanus* may contain, up to several thousand individuals. Such huge and dense colonies of genetically very similar organisms may pose specific problems to hygiene issues and pathogen defense [20]. Most interestingly, ants of this genus are exceptional in that they generally lack the so-called metapleural gland, which is known from other ants to be a major depository of antimicrobial compounds [21], [22]. Moreover, *Camponotus* and closely related genera harbor an obligate intracellular endosymbiont in specialized cells, the bacteriocytes, in their midgut, which need to be tolerated by the host's defense mechanisms [23], [24]. The recently published genome sequence of *C. floridanus* and subsequent bioinformatical analyses revealed the presence of two AMP genes encoding defensins [25], [26], which have significant similarities with defensins known from other ant species [12], [26], [27]. In addition to these *defensin* genes, a suppression subtractive hybridization approach also detected the presence of a *hymenoptaecin* gene in *C. floridanus* [28], which has not been annotated in the genome. In order to gain a better insight into the antimicrobial repertoire of *C. floridanus*, the present study aimed at the characterization of these antimicrobial peptides on the molecular level and comparison to AMPs encoded by other ant species. Most importantly, we show that in comparison to bee *hymenoptaecins* the *C. floridanus hymenoptaecin* gene is much longer and encodes a multipeptide precursor with structural similarities to apidaecin precursors from *A. mellifera* [17], the proteolytic processing of which possibly leads to a massive amplification of the antimicrobial response [13], [29].

## Materials and Methods

### Insect rearing and bacterial challenge

Founding queens of *C. floridanus* were collected in Florida near Orchid Island in August 2001 and were then kept in a climate chamber at Würzburg University as described before [30]. For pathogen challenge, *C. floridanus* major workers were injected with heat-killed *Serratia marcescens* (2×10^5^ cells/individual). At 24 h after injection, midgut and fat body were collected and kept in RNAlater (Ambion/Applied Biosystems, USA) until RNA preparation.

### DNA extraction and total RNA isolation

Total RNA from midgut and fat body of injected and naive major workers was extracted using TRIzol® Reagent (Invitrogen, Darmstadt, Germany) and purified through RNeasy mini kit columns (Qiagen, Hilden, Germany) with on-column DNase digestion (RNase-Free DNase Set, Qiagen) as described in the manufacturer's procedures. After purification, the RNA concentration of each sample was measured by the Nanodrop® spectrophotometer. RNA used in Northern blot analysis was additionally checked by PCR for gDNA contamination. *C. floridanus* genomic DNA was extracted from six larvae as described before [31].

### Sequencing of full-length cDNAs and genes

The complete sequences of the transcripts of interest were obtained by 3′ and 5′ RACE, performed with the SMART RACE cDNA Amplification Kit including the Advantage II PCR kit (Clontech, Heidelberg, Germany). For *defensin-1* and *hymenoptaecin*, the nucleotide sequences of the 3′- and 5′- primers (GSP1 and GSP2) were designed on the corresponding EST (GenBank Acc. No. for EST from *hymenoptaecin*: HS410972 and for EST from *defensin*: HS410966) obtained from a suppression subtractive hybridization (SSH) experiment [28]. For *defensin-2* RACE primers were designed according to the *C. floridanus* genome sequence. Primers used for RACE were Cfl_def-1-GSP1 and Cfl_def-1-GSP2 for *defensin-1*, Cfl_def-2-GSP1 and Cfl_def-2-GSP2 for *defensin-2* and Cfl_hym-GSP1 and Cfl_hym-GSP2 for *hymenoptaecin* (see Table S1). The first-strand cDNA used for 5′ and 3′-RACE were produced by using 1 μg of total RNA from *Serratia*-injected workers prepared for the SSH method, and using the primers provided in the kit. Amplification of the RACE products was carried out according to the manufacturer's instructions.

Oligonucleotide primers were then designed from the obtained RACE cDNA sequences and used for PCR amplification of the full length cDNAs und genes. Primers used were Cfl_def-1_flsF and Cfl_def-1_flsR for *defensin-1*, Cfl_def-2_flsF and Cfl_def-2_flsR for *defensin-2* and Cfl_hym_flsF and Cfl_hym_flsR for *hymenoptaecin* (see Table S1).

In each case resulting PCR-products were purified with the PCR Purification Kit (Qiagen), inserted into the plasmid vector pGEM (Promega) and transformed into *E. coli* DH5α cells (Invitrogen). The plasmids from several different clones were then extracted for sequencing with the UltraPrep Kit (Molzym, Bremen, Germany) according to the manufacturer's instructions. The sequences were generated by Seqlab (Sequence Laboratories Göttingen) with the vector primers M13 forward (5′-GTTTTCCCAGTCACGAC-3′) and M13 reverse (5′-CAGGAAACAGCTATGAC-3′).

### Hymenoptaecin probes for Northern and Southern blot analysis

In order to obtain specific *C. floridanus hymenoptaecin* probes for hybridization experiments, the inserts from two subtracted *hymenoptaecin* cDNA clones ([28], GenBank Acc. No. HS410972 and HS410975)) were amplified using PCR cycler and the Mol Taq PCR system (Molzym). Primers used were as follows: for the 270 bp *hymenoptaecin* 5′-probe: Cfl_hym_5′F and Cfl_hym_5′R; for the 270 bp *hymenoptaecin* repeat-probe Cfl_hym_repF and Cfl_hym_repR (see Table S1). PCR conditions were as follows: denaturation at 95°C for 3 min followed by 32 cycles of denaturation at 95°C for 15 s, annealing at 56°C for 15 s, and extension at 72°C for 1 min. PCR products were purified with the PCR Purification Kit (Qiagen).

### Northern blot analysis

For verification of *C. floridanus hymenoptaecin* full length mRNA, Northern blot analysis was performed. Total RNA from immune-challenged and naive workers (25 µg per lane) was separated on a 1.0% formaldehyde agarose gel and transferred onto a nylon blotting membrane (Amersham Hybond N^+^, GE Healthcare, UK). Membranes were prehybridized in roller bottles with 10 ml of Amersham Rapid-hyp Buffer (GE Healthcare, UK) for 30 min at 65°C. The *hymenoptaecin* 5′-fragment (see above) was radioactively labelled with 60 mCi of [α-^32^P]-dATP with the DecaLabel Kit (Fermentas, Germany) according to the manufacturer's specifications. The labelled cDNA probe was purified with Illustra Microspin S-200 HR Columns (GE Healthcare, UK). Membranes were rinsed and then washed two times for 15 min at 65°C in 50 ml of wash buffer (2× SSC, 0.1% SDS), sealed in saran wrap and exposed to a storage phosphor screen (GE Healthcare, UK) for 2 days. Screens were scanned on a Typhoon 9200 Variable Mode Imager (GE Healthcare, UK) with a resolution of 50 microns.

### Southern blot analysis

In order to characterize the *C. floridanus hymenoptaecin* gene locus, Southern blot analysis was performed. Digested DNA from immune-challenged and healthy workers (about 30 µg per lane) was separated on a 1.0% agarose gel and transferred onto a nylon blotting membrane (Amersham Hybond N^+^, GE Healthcare, UK). Membranes were prehybridized in roller bottles with 10 ml of hybridization buffer (0.5 M NaHPO_4_ (pH 7∶4), 1 mM EDTA, 0.7% SDS) for 30 min at 65°C. The *hymenoptaecin* repeat-fragment (see above) was labeled with Rediprime II DNA Labeling System (Amersham) according to the manufacturer's specifications. After addition of the heat-denatured probe (5 min at 95°C, cooled down on ice) hybridization was continued for 20 h at 62°C in a rotatory oven. Membranes were rinsed and then washed two times for 30 min at 65°C in 20 ml of wash buffer (0.04 M NaHPO_4_ (pH 7∶4), 1 mM EDTA, 0.5% SDS), sealed in saran wrap and exposed to a storage phosphor screen (GE Healthcare, UK) for 2 days. Screens were scanned on a Typhoon 9200 Variable Mode Imager (GE Healthcare, UK) with a resolution of 50 microns.

### Expression and purification of recombinant *Camponotus* hymenoptaecin

One of the putative mature *Camponotus* hymenoptaecins (*Cfl*-hym) was amplified by PCR with oligonucleotides Cfl_hymNdeI_F and Cfl_hymBamHI_R (see Table S1). The PCR product was inserted into pET-15b vector (Novagen, Merck KGaA, Darmstadt, Germany) at *Nde*I and *BamH*I sites with a thrombin cleavage site (LVPRGS) for subsequent removal of the N-terminal 6xHis-Tag. The resulting pET-15b-*Cfl*-hym was transformed into *E. coli* Rosetta 2(DE3)pRARE2 (Novagen, Merck KGaA) for protein expression. Expression of the recombinant *Cfl*-hym peptide was induced with 0.1 mM IPTG at an optical density of 0.5 at 600 nm. Cells were harvested after 5 h at 37°C and pelleted by centrifugation. Cell pellets were stored at −80°C until further purification.

Recombinant *Cfl*-hym peptide was purified from insoluble inclusion bodies under denaturing conditions. All purification steps were monitored by SDS-PAGE analysis using Tris-glycine gels. Thawed cell pellets were resuspended in resuspension buffer (100 mM NaH_2_PO_4_, 10 mM Tris-HCl, 8 M Urea, pH 8.0) and lysed by gently vortexing. Cellular debris was removed by centrifugation and the cleared lysate was mixed with 1 ml PerfectPro Ni-NTA Agarose (5 Prime GmbH, Hamburg, Germany). The lysate-resin mixture was then loaded on a column and washed twice with wash buffer (the resuspension buffer adjusted to pH 6.3). Finally, the fusion proteins were eluted by elution buffers (the resuspension buffer adjusted to pH 5.9 and subsequently pH 4.5). Suitable elution fractions were combined and carefully dialyzed to a final dialysis buffer concentration of 400 mM NaCl, 1 M Urea, 20 mM Tris-HCl, 20% glycerol at pH 7.4. In order to remove the N-terminal 6xHis-Tag, recombinant peptides were digested with thrombin using the Thrombin Cleavage Capture Kit (Novagen, Merck KGaA) according to the manufacturer's instructions. The cleaved peptide was concentrated by ultrafiltration using Amicon Ultra-4 centrifugal filter devices (Millipore, Schwalbach, Germany). For buffer exchange the concentrated protein solution was diluted in dialysis buffer (400 mM NaCl, 1 M Urea, 20 mM Tris-HCl, 20% glycerol at pH 7.4) to a volume of 4 ml and subsequently concentrated again as described above. This procedure was repeated four times. Protein concentrations were measured using the method of Bradford with bovine serum albumin as standard.

### Inhibition zone assays

Inhibition zone assays were performed in order to determine antibacterial activity of the purified *Cfl*-hym peptide. Microorganisms used in the inhibition zone assays were *E. coli* D31 [32] as a gram-negative bacterium and *Bacillus subtilis* as a gram-positive bacterium. In each case 3–4×10^5^ bacteria were diluted in 3 ml preheated Luria broth (LB) containing 0.75% agarose. The mixture was spread out evenly on preheated LB agar plates. After settling, blanc discs (Oxoid, Thermo Fisher Scientific, Schwerte, Germany) were put on the agar plates and 4.5 nmol *Cfl*-hym peptide (in dialysis buffer) were applied on top. Furthermore dialysis buffer alone was applied as a negative control and 4 µg kanamycin as a positive control. The plates were incubated at 37°C overnight. On the next day the clear zone of inhibition was documented by photography.

### Bioinformatical prediction of proteins in ant genomes

Gene prediction was performed for the published genomes of the ant species C. floridanus, Atta cephalotes, Harpegnathos saltator, Pogonomyrmex barbatus, Solenopsis invicta, Linepithema humile, and Acromyrmex echinatior using the gene prediction pipeline maker (version 2.11-beta) [33]. Therefore, the genomic contigs were prefiltered by BLASTX (version 2.2.24). All contigs having hits against published hymenoptaecin or defensin proteins were used for the gene prediction pipeline. The identified C. floridanus cDNA sequences and all available protein sequences from A. mellifera or other ants were used as EST and protein evidence for the gene predictions. Augustus with its Nasonia model was used for de novo gene predictor [34]. The obtained gene predictions were manually curated.

### Phylogenetic tree reconstruction for mature hymenoptaecin peptides

The sequences for the cDNAs and proteins resulting from the gene prediction and the real sequences obtained from *C. floridanus* were analyzed by the ProP server (version 1.0) [35]. All cDNAs were fragmented according to the cleavage sites predicted by ProP. All obtained single domain cDNA fragments were aligned by translator [36] with default settings and the resulting alignment was cleaned by Gblocks [37] with the default settings from the translatorX website. The phylogenetic tree was reconstructed by PhyML (version 3.0.1) [38] under the GTR+I+G+F model with 100 bootstrap replicates as implemented in seaview (version 4.3.0) [39]. Branches with a bootstrap support below 40 were combined using iTOL [40] and the tree was drawn with the software FigTree (version 1.3.1).

### Phylogenetic tree reconstruction and tree reconciliation for defensin peptides

For *C. floridanus* and *S. invicta* two defensin peptides were predicted. Therefore, we added *Ixodes scapularis* to the sequence set as outgroup (GenBank Acc. No.: XP_002436104.1) and the two *A. mellifera* defensins defensin-1 (GenBank Acc. No.: NM_001011616.2) and defensin-2 (GenBank Acc. No.: NM_001011638.1). The whole sequence set was aligned by MUSCLE (version 3.8.31) [41]. The tree was reconstructed by BioNJ [42]. Therefore, the observed amino acid frequencies were used. Branches with a bootstrap support below 40 were combined using iTOL [40]. The species tree for tree reconciliation was derived from Brady *et* *al*. 2006 and Gadau *et* *al.* 2012 [43], [44]. For tree reconciliation the software Notung (version 2.6) [45], [46] was used. The gene tree and the reconciled tree were drawn using FigTree (version 1.3.1).

## Results

### Cloning and sequence analysis of a hymenoptaecin encoding cDNA of *C. floridanus*

**Figure 1:**
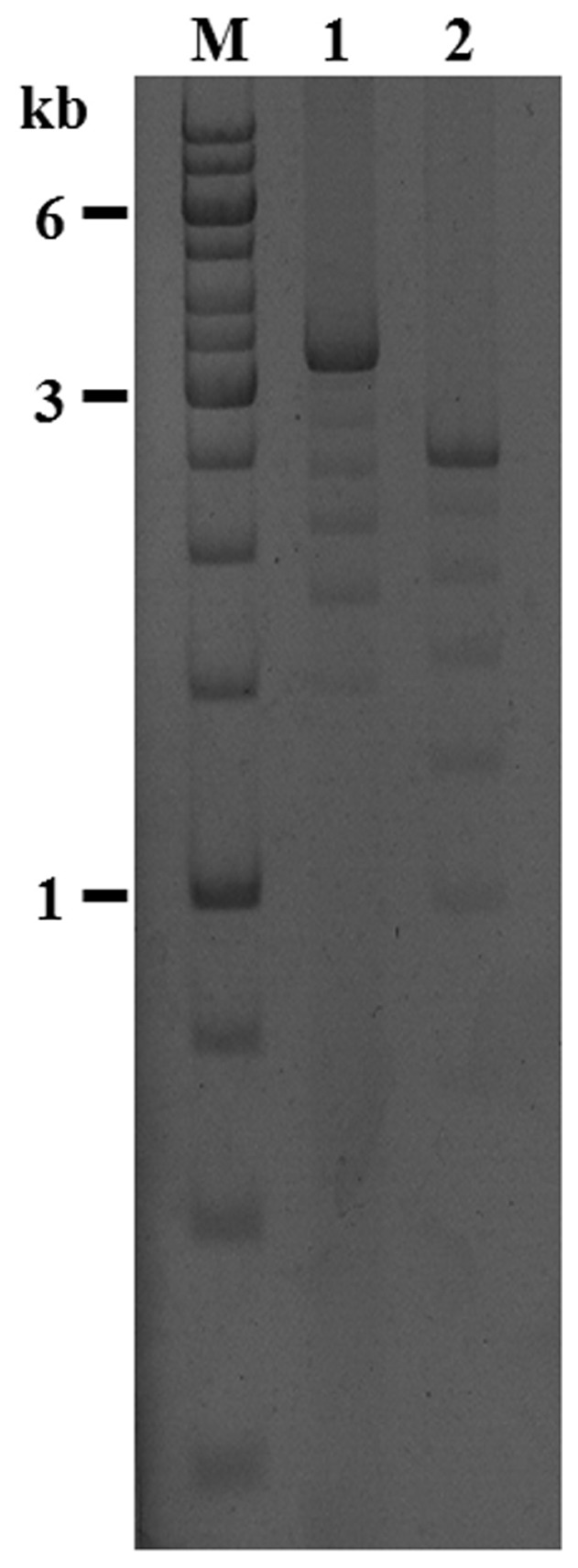
PCR amplification of full length *hymenoptaecin* gene and cDNA. The PCR-products from gDNA (lane 1) and cDNA (lane 2) were separated on a 1.2% agarose gel alongside molecular size markers (lane M, GeneRuler 1 kb DNA Ladder, Fermentas) and analyzed with EtBr staining. The major bands correspond to the full length *hymenoptaecin* gene- (3356 bp lane 1) and cDNA-product (2536 bp, lane 2). The minor bands are technical artefacts with variable repeat numbers caused by the tandem repeats.

**Figure 2:**
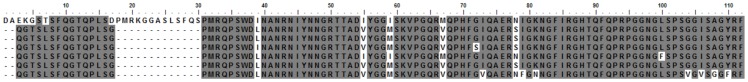
Alignment of HLD (hymenoptaecin-like domain) and all HDs (hymenoptaecin domains) from the same *C. floridanus* hymenoptaecin multipeptide precursor protein. Grey boxes indicate conserved residues. The insertion in the hymenoptaecin-like domain (top) is clearly visible.

The search for immune inducible genes in *C. floridanus* by a SSH approach revealed the presence of a cDNA encoding a homologue of hymenoptaecin, an AMP known from other hymenopteran species [28]. The subsequent attempts to define the 5′- and 3′ends of the cDNA resulted in complex patterns. As expected the 5′RACE of the *hymenoptaecin* cDNA revealed one product. However, the 3′RACE resulted in several products of different length. Further investigation of these products revealed that all were *hymenoptaecin* derived 3′RACE products with an identical 3′UTR and a poly-A tail. The various molecules with different length resulted from a 309-nucleotide sequence which was repeated several times but in different copy number in the different amplification products. To confirm the existence of these deduced cDNAs the full length *hymenoptaecin* cDNAs were amplified with primers binding near the 5′and 3′-ends. Several clones with insert size varying from 681 bp to 2536 bp were identified and analyzed (Fig. 1). All of the examined cDNA sequences contained a constant 5′- and 3′end, embracing a region of variable length containing one to six copies of the 309-nucleotide repeat sequence. All putative precursor proteins deduced from the different amplification products are composed of a signal peptide of 19 amino acids (aa), a propeptide of 26 aa and a mature peptide region of differing length in dependence of the repeat number. The latter seems to be further processed into multiple mature AMPs in accordance with the presence of proprotein convertase cleavage sites Arg (R)/Lys (K), as predicted by ProP 1.0 [35]. Several different hymenoptaecin peptide variants could be deduced from the analyzed ESTs. Considering all obtained cDNA sequences and the size of the major product, we deduced a major *hymenoptaecin* mRNA (GenBank Acc. No.: HQ315784) of 2536 bp containing an ORF of 2373 bp (781 aa) corresponding to six repeats of the putative mature peptide sequence. Each of the repeated units consists of a coding sequence for the mature hymenoptaecin peptide, preceded by coding regions for a spacer sequence (EAEP) and a putative proprotein cleavage site (RR or KR) (Fig. 2). Further analysis of the deduced mature peptides showed that all putative hymenoptaecin peptides are 97 aa long and start with a glutamine (Q) at their N-terminus. Only the first peptide of each precursor, the so called hymenoptaecin-like peptide, displayed an exception consisting of 108 aa due to an N-terminal insertion (Fig. 2) and starting with a glycine (G). In sum, the domain composition of the *Camponotus* hymenoptaecin resembled the multipeptide precursor structure of bee apidaecins [29].

### Genomic organization of *hymenoptaecin*

**Figure 3:**
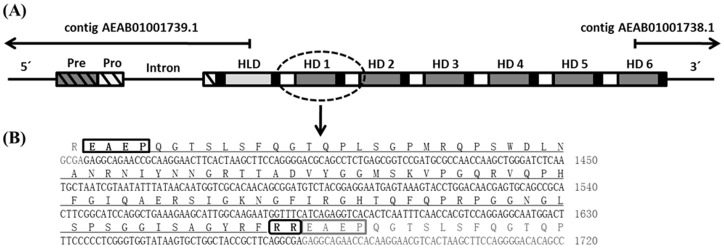
Structure of the *C. floridanus hymenoptaecin* gene locus. (A) Schematic structure of the *hymenoptaecin* gene containing a single intron within the region coding for the hymenoptaecin propeptide. The deduced multipeptide precursor peptide consists of a signal-sequence (Pre, grey hatched box) and a pro-sequence (Pro, white hatched box), followed by a hymenoptaecin-like domain (HLD, light grey box) and six repeated hymenoptaecin domains (HD 1–6, dark grey boxes). The hymenoptaecin domains are flanked by the two putative processing sites EAEP (white boxes) and RR (black boxes). (B) The nucleotide and deduced amino acid sequence of a hymenoptaecin repeat unit are shown and the putative processing sites are boxed.

**Figure 4:**
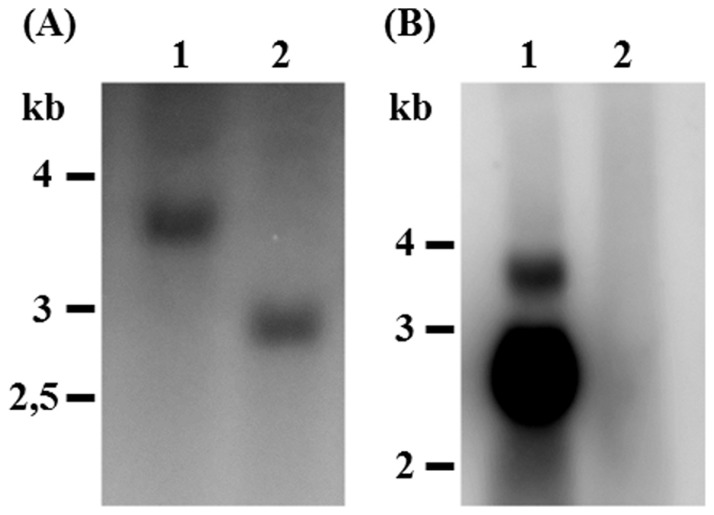
Southern blot (A) with *C. floridanus* genomic DNA using a ^32^P-labelled *hymenoptaecin* fragment corresponding to one of the repeats as a probe. Genomic DNA (35 µg per lane) was digested with *EcoR*I (lane 1) and with *Bsu15*I (lane 2), separated by gel electrophoresis and hybridized with the above mentioned DNA fragment. Northern blot (B) with total RNA of *C. floridanus* using a ^32^P-labelled cDNA fragment corresponding to the 5′end of the *hymenoptaecin* gene as a probe. Total RNA (25 µg per lane) was isolated from midgut and fat body of major workers injected with heat-killed *Serratia marcescens* (2×10^5^ bacteria/ant) in the haemocoel (lane 1) or untreated animals (lane 2). The major band corresponds to the spliced mature transcript, while the minor band very likely is the unspliced precursor. The position of molecular size markers is indicated on the left side of each figure. All hybridizing bands have the expected molecular size.

As described above, by comparison of all possible repeat versions from the sequenced *hymenoptaecin* cDNAs, we found a high number of different deduced hymenoptaecin peptide variants. Therefore we addressed the question whether this diversity was caused by the existence of multiple *hymenoptaecin* genes in the *C. floridanus* genome or by alternative splicing of large transcripts from a single gene. To solve this question, we amplified the *hymenoptaecin* gene(s) by PCR using conditions for the amplification of large fragments using the Cfl_hym_fls forward and reverse primers (see Table S1). Similar to the amplification of the *hymenoptaecin* cDNAs the PCR with gDNA yielded a ladder of amplified products ranging from 1500 bp to 3356 bp in length (Fig. 1). Comparing gDNA to mRNAs, we located one phase 0 intron of 820 bp in size, which is present after the codon coding for histidine number 39 (Fig. 3). As no other introns were found, the observed variation in repeat numbers cannot be explained by alternative splicing of exons coding for the repeats. Therefore, we investigated the question, whether hymenoptaecin variants may be encoded by a multigene family. In Southern Blot analysis of *Bsu15*I-digested DNA from multiple insects a fragment of about 2.9 kb in size hybridized with a cDNA probe derived from the repeat sequences (Fig. 4). The size of this fragment was consistent with that expected from digestion of the main PCR-product containing six repeats. Since no other signal was found, this result suggests that hymenoptaecin is encoded by a single gene which harbours six repeated sequence motifs (Fig. 3). This is also confirmed by Northern Blot analysis which resulted in a major band of the expected size of the mature transcript and a larger minor band which probably represents the unspliced primary transcript, since its size perfectly matches the predicted size (Fig. 4). In sum, our data suggest the existence of a single *C. floridanus hymenoptaecin* gene (GenBank Acc. No.: HQ315784) and we suppose that the above described variable repeat numbers after PCR amplification were a technical artefact caused by the tandem repeats.

### Antibacterial activity of recombinant *C. floridanus* hymenoptaecin

**Figure 5:**
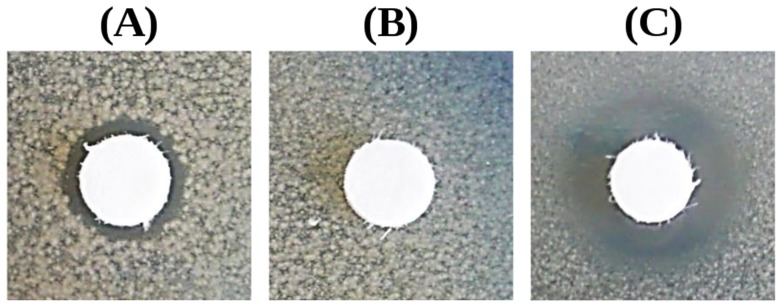
Antibacterial activity of 4.5 nmol recombinant *Cfl*-hym peptide (A) against *E. coli* D31. Dialysis buffer alone (B) was applied as a negative control and 4 µg kanamycin (C) as a positive control.

The 6xHis-tagged hymenoptaecin fusion protein (*Cfl*-hym) of 12.7 kDa was expressed in an insoluble form in *E. coli* Rosetta 2(DE3)pRARE2 (Novagen, Merck KGaA) and thus had to be purified under denaturing conditions. In order to keep the peptide soluble after digestion and refolding steps, dialysis buffer (containing 1 M Urea and pH 7.4) was most suitable. The antibacterial activity of the purified *Cfl*-hym peptide was tested against gram-negative *E. coli* D31 and gram-positive *Bacillus subtilis* with inhibition zone assays. Dialysis buffer alone was included as a negative control. Under the experimental conditions *Cfl*-hym was only active against *E. coli* D31 (Fig. 5).

### Phylogenetic analysis of hymenoptaecin peptides and comparison of the different hymenoptaecin multipeptide precursors

**Figure 6:**
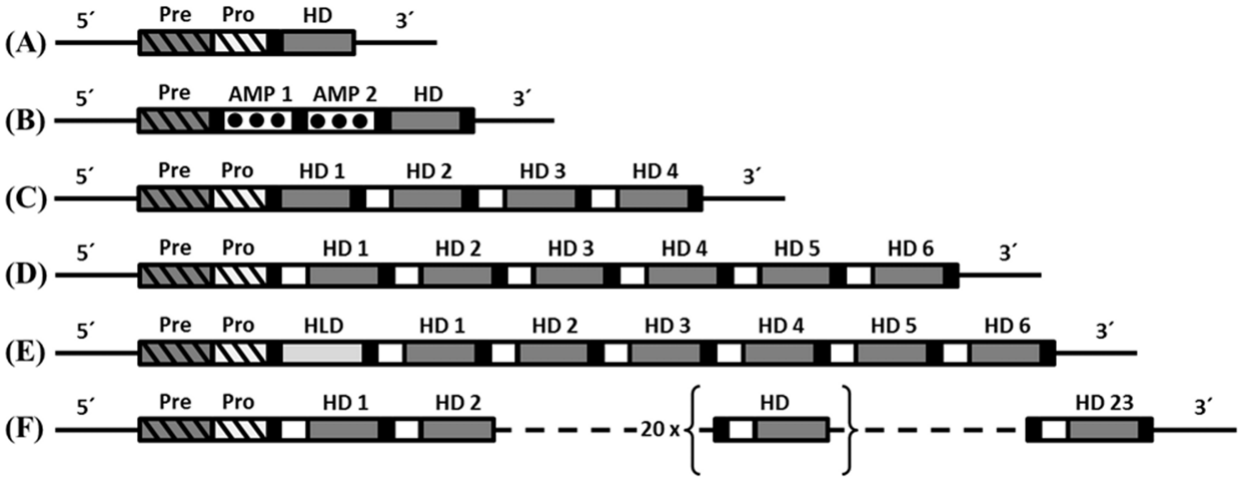
Schematic structure of the hymenoptaecin precursors from different hymenopteran species: A) *Apis mellifera* (GenBank Acc. No.: NP_001011615) or *Bombus ignitus* (GenBank Acc. No.: ACA04900); B) *Nasonia vitripennis*: (GenBank Acc. No.: NP_001165829 XP_001607881); C) *Harpegnathos saltator* 1 (GenBank Acc. No.: EFN79831); D) *Harpegnathos saltator* 2 (GenBank Acc. No.: EFN79832); E) *Camponotus floridanus* (GenBank Acc. No.: HQ315784); F) *Acromyrmex echinatior* (hymenoptaecin multipeptide precursor deduced from genome draft). The various domains are marked as follows: signal-sequence (grey hatched box), pro-sequence (white hatched box), hymenoptaecin-like domain (HLD, light grey box), hymenoptaecin domains (HD 1–6, dark grey boxes), proline-rich AMP-like peptide (AMP 1–2, white dotted boxes). The hymenoptaecin domains are flanked by the putative processing sites EAEP (EANP for *Harpegnathos*) (white box) and RR (or RxxR) (black box).

The recently established genome sequences of six different ant species revealed the presence of at least one AMP belonging to the hymenoptaecin family in each ant species [25], [47], [48], [49], [50]. For some of the ant species hymenoptaecin proteins were annotated. First we used these predicted proteins and found unusual domain compositions. Additionally, some of the proteins show a lack of crucial elements like recognition sequences for signal- and propeptides. Therefore, we investigated the predicted proteins on genome level using the published genome drafts. On genome level we could identify the problems which lead to the wrong prediction results. The sequence region, which apparently codes for the hymenoptaecin peptide(s) in the species *Atta cephalotes*, *Linepithema humile*, *Pogonomyrmex barbatus* and *Solenopsis invicta* seems to span contig boundaries, which were filled with N's during the scaffolding. This is based on the general problem to assemble short read sequences from next generation sequencing methods through regions with repetitive sequence elements. Three different hymenoptaecin precursor proteins were predicted for the genome of the ant species *Acromyrmex echinatior* (GenBank Acc. No.: EGI65977, EGI65978 and EGI65979) [48]. However, from the analysis of the *A. echinatior* genome we could deduce one putative hymenoptaecin multipeptide precursor, which combines the three predicted ones due to missing stop codons between the predicted proteins. The obtained hymenoptaecin peptide region of this precursor is extremely long and codes for 23 putative mature hymenoptaecin peptides (Fig. 6F). The two annotated hymenoptaecin precursor proteins from *Harpegnathos saltator* seemed to be plausible and contain either four (GenBank Acc. No.: EFN79831) or six (GenBank Acc. No.: EFN79832) mature AMPs, respectively.

**Figure 7:**
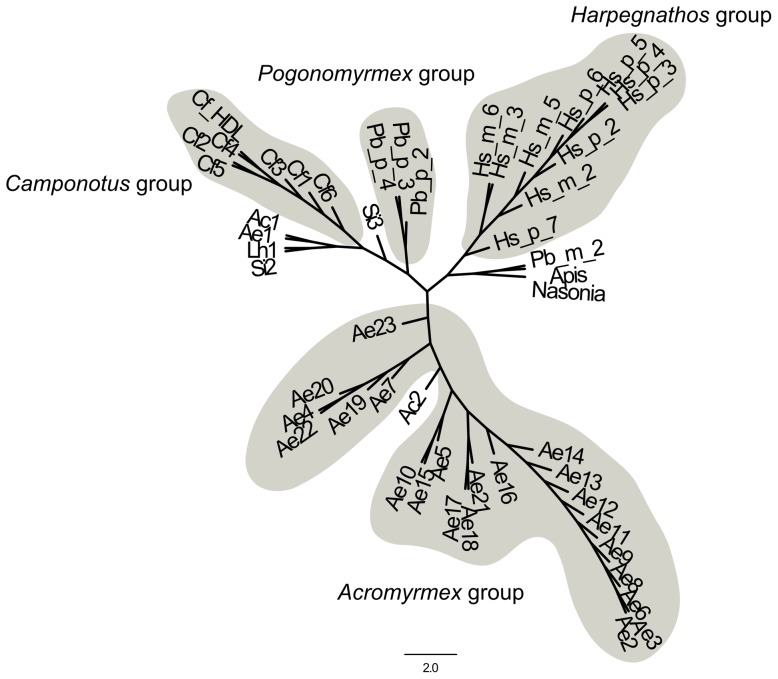
Phylogenetic analysis of hymenoptaecin domains from different ant species. Shown is the unrooted tree of the single domains of the hymenoptaecins of the ant species, *N. vitripennis*, and *A. mellifera*. The proteins were cleaved at the sites predicted by ProP, followed by the alignment by translatorX. The tree was reconstructred by PhyML with a GTR+I+G+F model with 100 bootstrap replicates. The domains of the species with a complete hymenoptaecin protein form clades and are named as groups according there genus name and are indicated by their grey background. The domains which are outside these groups result from missing data from the predicted genes. The gene models are incomplete due to long N-stretches in the genomic sequences based on the scaffolding process. Nevertheless, the distinct groups formed by the complete proteins suggest an intra-species mechanism for the accumulation of the single domains.

Fig. 6 shows a schematic comparison of the domain structures of hymenoptaecins from different hymenopteran species. Interestingly, the deduced hymenoptaecin precursor proteins from ant species are all multidomain proteins with remarkable similarities to the multipeptide precursor of *C. floridanus* hymenoptaecin. They have varying numbers of hymenoptaecin domains (HDs), which are all flanked by the putative spacer region EAEP (EANP for *Harpegnathos*) and processing sites RR (or RxxR, as predicted by ProP 1.0). Our phylogenetic analysis of the hymenoptaecins based on the existing set of proteins suggests an intra-species accumulation of the single domains within the proteins (Fig. 7, see also Fig. S1).

### Cloning and sequence analysis of *C. floridanus* defensins

**Figure 8:**
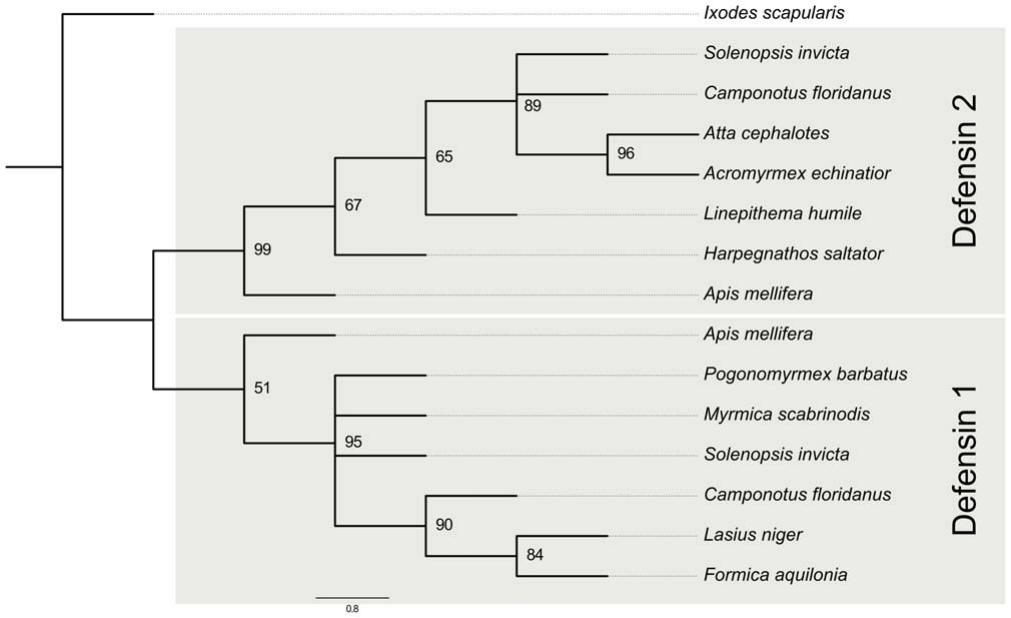
Phylogenetic analysis of defensins from different ant species. All defensin sequences were aligned by MUSCLE and a BioNJ-tree with 100 bootstrap replicates was calculated. Branches with a bootstrap support below 40 were removed. Other bootstrap values are indicated. The genes for the gene tree were generated using the gene prediction pipeline maker and hand curated. The gene tree was rooted at the defensin from *Ixodes scapularis* (GenBank Acc. No.: XP_002436104.1). The *A. mellifera* defensin-1 forms a clade with proteins formerly described as defensin-2, which gives a first indication that they could be renamed accordingly. However, some of the proteins form a clade with the *A. mellifera* defensin-2. Moreover, two species *S. invicta* and *C. floridanus,* own both defensins. Therefore, we suggest a duplication event at the LCA of *A. mellifera* and the ant species.

The SSH approach performed to identify immune inducible genes as well as the screening of the genome sequence resulted in the identification of two different sequences coding for defensin-like AMPs in *C. floridanus*. Phylogenetic analysis allocated these sequences as homologues to defensin-1 and defensin-2 from *A. mellifera* (Fig. 8).

**Figure 9:**
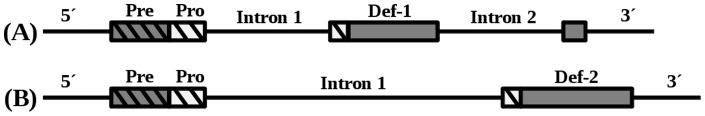
Schematic structure of the *defensin* genes from *C. floridanus*. (A) The gene encoding defensin-1 (GenBank Acc. No.: JN989495) is composed of three exons and two introns. The first intron is located within the region coding for the propeptide and the second is located within the region coding for the mature defensin peptide. (B) The gene encoding defensin-2 (GenBank Acc. No.: JQ693412) contains only one intron, which is also located within the propeptide coding region. Both deduced precursor peptides consist of a signal-sequence (Pre, grey hatched box) and a pro-sequence (Pro, white hatched box), followed by the mature defensin peptide (Def, grey box).

5′RACE and 3′RACE of the *defensin-1* cDNA suggested a full length mRNA sequence of 535 bp (GenBank Acc. No.: JN989495), which was verified by amplification with primers near the 5′- and 3′-ends. The deduced *C. floridanus* defensin prepropeptide is 102 amino acids (aa) long, including a signal peptide of 17 aa and a propeptide of 40 aa, followed by a mature peptide of 44 aa, as predicted by ProP 1.0 [35]. The subsequent cloning of the corresponding *defensin* gene revealed the presence of three exons (64, 229 and 13 bp) and two introns (399 and 360 bp). The first intron is a phase 1 intron, which is located within the codon of glutamic acid number 22. The second intron is a phase 2 intron at the alanine residue number 98 following a so-called CXC motif characteristic for defensins (Fig. 9A).

In contrast, the *defensin-2* gene contains only two exons (97 and 194 bp), which are separated through one phase 1 intron (1043 bp) located within the codon of threonine number 33 (Fig. 9B). The full length mRNA sequence of *defensin-2* (GenBank Acc. No.: JQ693412) is 1238 bp long and encodes a prepropeptide of 97 aa consisting of a signal peptide of 18 aa, a propeptide of 36 aa and a mature defensin peptide of 43 aa, as predicted by ProP 1.0 [35].

### Phylogenetic analysis of defensin peptides

**Figure 10:**
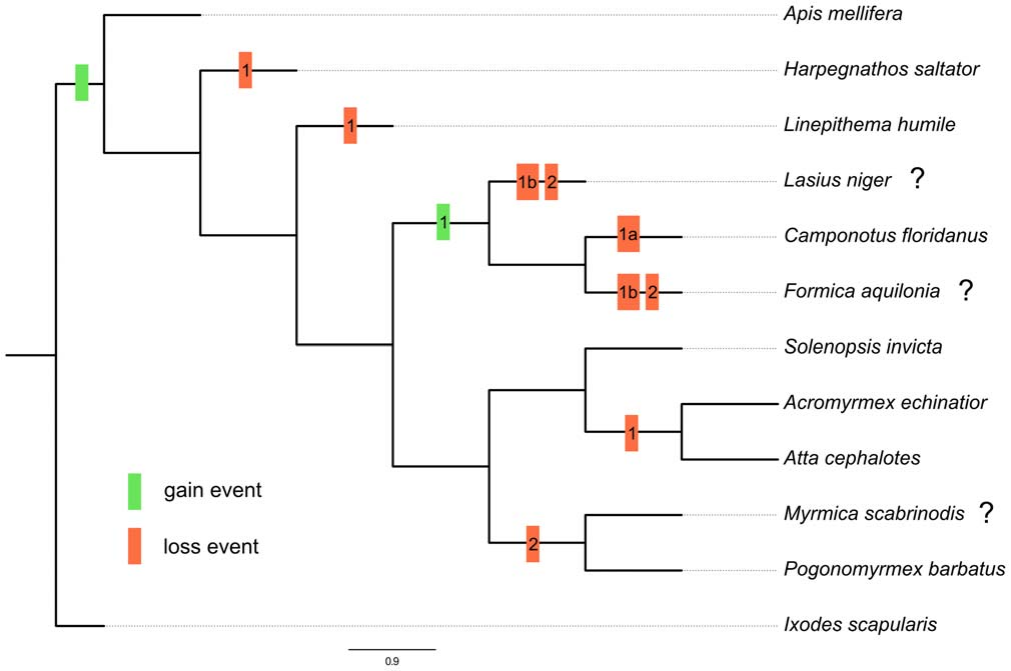
Reconciled species tree of ant defensins. This tree was generated by Notung. The ant species *L. niger* (GenBank Acc. No.: ACB46517.1), *Myrmica scabrinodis* (GenBank Acc. No.: ACB46524.1), and *F. aquilonia* (GenBank Acc. No.: Q5BU36.1) are examples for the sequences generated by [12], [27]. For these species no whole genome is available, which is indicated by the question mark behind the species. The gain and loss events are indicated by green and red boxes. The gain event at the LCA of *A. mellifera* and the ant species generated the defensin-1/2 peptide. The LCA of *L. niger*, *C. floridanus* and *F. aquilonia* had an additional duplication event of its defensin-1 peptide, but the species lost either their defensin-1a or their defensin-1b.

The comparison of the deduced amino acid sequences of the *C. floridanus* defensins with other ant defensins suggests a duplication event in the last common ancestor (LCA) of the bee *A. mellifera* and the ant species (Fig. 10). Nevertheless, almost all ant species have lost one of their defensin proteins. Only *C. floridanus* and *S. invicta* possess two defensins. Moreover, most of the proteins forming a clade with the *A. mellifera* defensin-1 were formerly described as defensin-2 homologues. Therefore, these proteins should be renamed to defensin-1 (Fig. 8, see also Fig. S2). Furthermore, an additional duplication event of defensin-1 peptide seems to have taken place in the LCA of *Lasius niger*, *C. floridanus* and *Formica aquilonia*, due to the grouping of the defensin-1 proteins in the phylogenetic tree (Fig. 8), which does not represent the species tree (Fig. 10). Nevertheless, the species lost either their defensin-1a (*C. floridanus*) or their defensin-1b (*L. niger* and *F. aquilonia*; Fig. 10).

## Discussion

AMPs are essential components of the insect immune system. Here we describe the identification and initial characterization of AMP genes of the ant *C. floridanus* encoding two defensins and a hymenoptaecin. Our results, taken together with the genome sequence of this social insect, indicate that these are the only three genes in *C. floridanus* encoding AMPs. Thus, similar to *A. mellifera*, a tendency to reduce the immune gene repertoire was suggested for ant species possibly due to hygiene measures on the colony level [13], [20].

### Characterization of *C. floridanus* hymenoptaecin

In the recently published genome sequence of *C. floridanus* the *hymenoptaecin* gene escaped detection possibly due to sequencing problems of this gene carrying multiple direct repeats [25]. However, two contigs (AEAB01001738.1 and AEAB01001739.1) were found which harbour the 5′- and 3′- ends of the gene (Fig. 3). Since no other contigs encoding DNA sequences resembling the *hymenoptaecin* gene were discovered, the genomic data also supports the existence of a single *hymenoptaecin* gene, thus confirming that the above described variable repeat numbers after PCR amplification were a technical artefact caused by the tandem repeats. The characterization of the hymenoptaecin revealed a very peculiar modular composition of the deduced peptide(s) as compared to hymenoptaecins of other Hymenoptera. The hymenoptaecins known from other hymenopterans such as *A. mellifera* [9] and *B. ignitus* [10] show significant sequence homology to the hymenoptaecin domains repeated several times in the multipeptide precursor of the *C. floridanus* hymenoptaecin, suggesting structural and functional similarities. The *A. mellifera* hymenoptaecin is 93 aa long, including a 2-pyrrolidone-5-caboxylic acid at the N-terminus, which is derived from glutamine [17]. With the exception of the first so-called hymenoptaecin-like domain, the six deduced mature *C. floridanus* hymenoptaecins are 97 aa long and all start with a glutamine residue. Therefore, an amino-terminal blocking by forming 2-pyrrolidone-5-carboxylic acid is very likely also for the *C. floridanus* hymenoptaecin peptides. One of the putative mature hymenoptaecin peptides was overexpressed in *E. coli* and shown to exhibit moderate antibacterial activity. The possibility of an amino-terminal blocking might further increase the antibacterial potency of *Cfl*-hym peptides.

In contrast to the *A. mellifera* hymenoptaecin the *C. floridanus* hymenoptaecin has a complex precursor organization. A comparable precursor organization is known for the *N. vitripennis* hymenoptaecin, which encodes three AMP-like peptides, including one with similarity to the hymenoptaecin domains of *C. floridanus* [19]. Overall, the *C. floridanus* hymenoptaecin precursor structure is more similar to the multipeptide precursor structure of apidaecins, consisting of several repeated units [29]. As for apidaecin precursors we assume that the mature hymenoptaecin peptides are released by a three step mechanism, which is similar to maturation procedure of the yeast alpha-mating factor, since the repeats are flanked by repeating -X-A- (or -X-P-) sequences [51]. The initial processing is probably mediated by the KEX2-encoded endoprotease, which cuts at the C-terminus of the basic dipeptides Arg/Lys (RK) or Arg/Arg (RR) [52]. The next step is the C-terminal maturation via the KEX1-encoded carboxypeptidase, which removes both basic residues [53]. The last step is the N-terminal maturation of the spacer-mature peptides by a dipeptidyl aminopeptidase that removes E/D-A/P dipeptides [51]. Homologues of the respective enzymes are present in *C. floridanus* (GenBank Acc. No. EFN61704 to CAA96915 (E-value: 3E-35), EFN64345 to CAA96143 (E-value: 7E-94) and EFN67964 to NP_014862 (E-value: 5E-55)).

Despite the similarities in the multipeptide precursors, the *C. floridanus hymenoptaecin* differs from the *A. mellifera apidaecin* with regard to the gene structure. The latter one consists of several exons, each encoding a functional and distinct apidaecin peptide [13]. In contrast the hymenoptaecin mature peptide regions are encoded by a single exon only. This intronless gene structure prohibits the possibility of generating different transcripts by splice variation. Nevertheless, the multipeptide precursor structure of the *Camponotus hymenoptaecin* gene would allow the amplification of the antibacterial response despite the presence of only a single gene, as it was already suggested for apidaecins [29]. Furthermore, we also find surprisingly high sequence variability in our pooled samples. This high level of individual sequence variation has also been described for apidaecin exons from different bees [13], [29].

### Phylogenetic analysis of *hymenoptaecin* peptides

The genome sequences of other ant species [25], [47], [48], [49], [50] revealed the presence of at least one gene locus encoding a hymenoptaecin precursor with similar domain structure as the *C. floridanus* hymenoptaecin. The presence of such multidomain *hymenoptaecins* in all ant species indicates an ancient origin of this gene structure early in the evolution of ants. However, the genome sequences indicate problems during the assembly and the gene prediction step of the genome projects and only a few sequences seem to be complete. All investigated complete hymenoptaecin peptide regions are encoded by a single exon only, which is evidence for exon duplication [54]. Our preliminary phylogenetic analysis of mature hymenoptaecin peptides suggests that the duplication event occurred independently in each species after separation (Fig. 7). Ongoing studies will reveal the full length hymenoptaecin precursor sequences from other ant species by direct sequencing and will deliver insights into the evolutionary history of the hymenoptaecin protein family in ants.

### Phylogenetic analysis of ant defensin peptides

Bioinformatical prediction of defensins revealed the presence of at least one *defensin* gene in all investigated ant genomes with homology to *defensin-1* or *defensin-2* from *A. mellifera* (Fig. 8). We show that *C. floridanus* and *S. invicta* encode both *defensin* genes. Therefore, we suggest that the LCA of the ants and *A. mellifera* encoded both *defensins*. Based on the assumption that the genomes of *L. niger, F. aquilonia,* and *M. scabrinodis* contain only the published *defensin* genes, the reconciled gene tree (Fig. 10) exhibits many gain and loss events. Multiple duplication and loss events indicate a high adaptive potential and evolutionary plasticity of the antimicrobial peptides in ants. The *C. floridanus* mature defensin-1 and defensin-2 peptide sequences are well conserved with other ant defensins. However, the *defensin-1* gene comprises three exons and two introns, in contrast to other characterized ant *defensin* genes [12], [27], which have two exons and one intron. Interestingly, a similar intron-exon composition is also known for other hymenopteran *defensin-1* genes, e.g. from *A. mellifera* [16], *B. ignitus* [10] and *N. vitripennis* [11], while the *Drosophila defensin* gene does not carry any intron at all [55]. In contrast to other insect defensins, the bee defensin-1 has an extra stretch of 11 amino acids at its C-terminus, which encodes an additional C-terminal α-helical domain [9]. The *C. floridanus* defensin-1 has a short C-terminal extension of three amino acids in length. The precursors of the bee defensins have an extra amino acid, a glycine (G), at their C-termini, which seem to be amidated as suggested in the mature *A. mellifera* defensin [9]. As the deduced *C. floridanus* defensin-1 also ends with a G, it mayas well be amidated. According to this the mature *C. floridanus* defensin-1 is 3 amino acids longer than all other known ant defensins and it is so far the only known ant defensin which has an additional exon that is lacking from most other insects [56]. Further investigations will reveal, if this C-terminal extension can also be found in defensins from other ant species or if it is a special feature of *C. floridanus*.

### Conclusions

The data reported here in combination with the recently published ant genome sequences indicate that the hypothesis of a reduced immune gene repertoire in social insects cannot easily be adopted for ant species. The genome drafts of *C. floridanus* and *H. saltator* [25] suggest indeed a comparable low number of genes encoding AMPs. However, this low number may to a certain extent be counteracted by the amplification of hymenoptaecin domains which are encoded as large precursor proteins with multiple bioactive domains. Sequence variations in the mature peptides may also lead to diversification of the immune response. Furthermore, *P. barbatus* even has more AMP genes than *A. mellifera* [50]. Detailed analyses of the complete antimicrobial repertoire from different ant species will deliver a better classification of the individual defense capabilities of these social insects.

## Supporting Information

Figure S1 **Alignment of hymenoptaecin domains from different ant species.** The cDNAs of the ants were fragmented according to the cleavage sites predicted by ProP. Afterwards, all cDNA fragments were aligned by translatorX with default settings by muscle and the resulting alignment was cleaned by Gblocks with the default settings from the translatorX website.
(TIFF)

Figure S2 **Alignment of defensin peptides from different ant species.** The predicted peptide sequences for the seven ant species, the defensin-1 and defensin-2 of *Apis mellifera* (NM_001011616.2, NM_001011638.1), and the defensin of *Ixodes scapularis* (XP_002436104.1) were aligned by muscle with default settings.
(TIFF)

Table S1 Primers used for characterization of *C. floridanus* hymenoptaecin and defensins.
(PDF)
