# Supplementary figures and images for: Molecular Characterization of Antimicrobial Peptide Genes of the Carpenter Ant Camponotus floridanus
Source: PLoS One. 2012 Aug 9;7(8):e43036. doi: 10.1371/journal.pone.0043036 (PMC3415428; doi:10.1371/journal.pone.0043036)

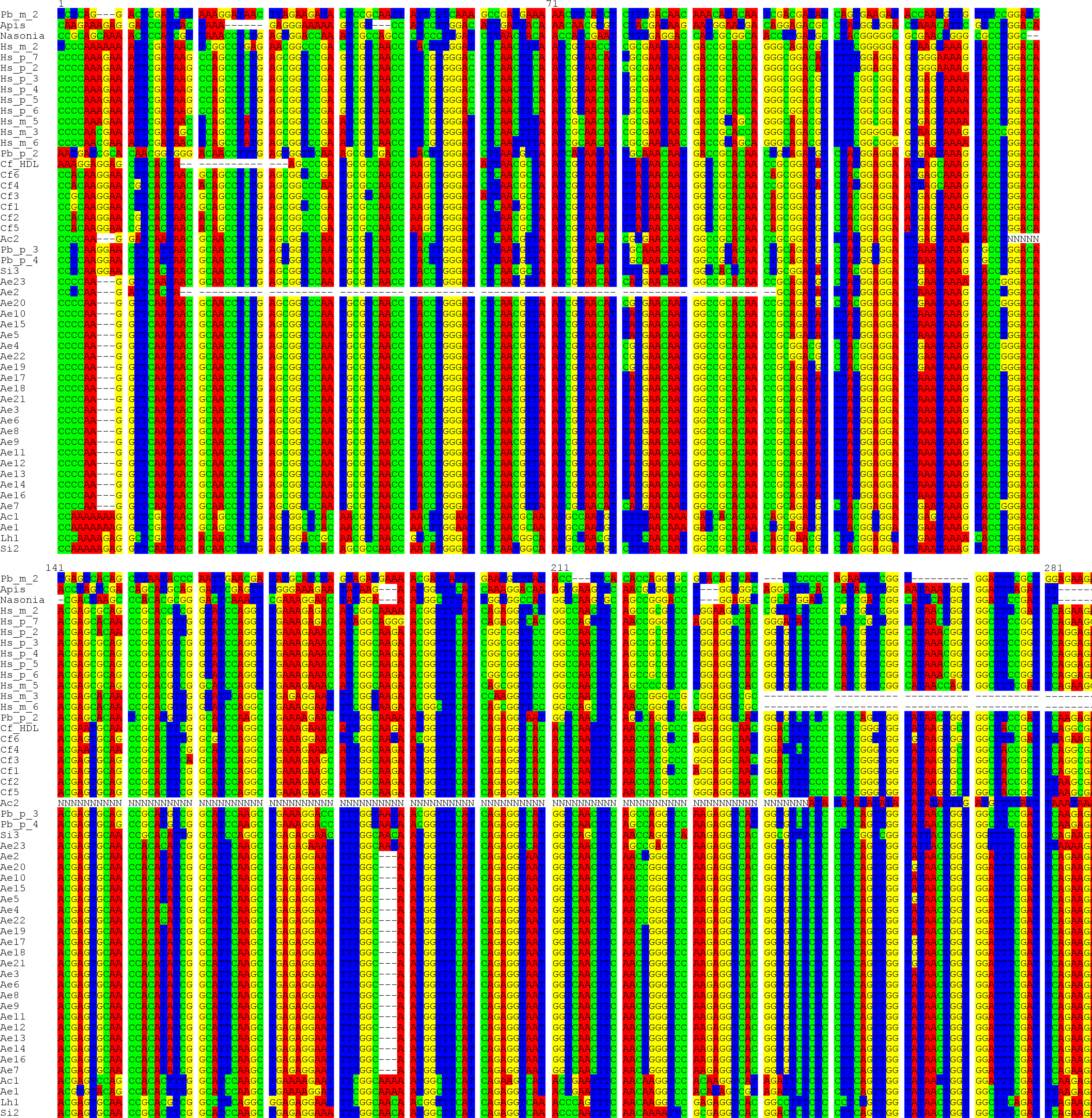

Supplement: Figure S1 — Alignment of hymenoptaecin domains from different ant species. The cDNAs of the ants were fragmented according to the cleavage sites predicted by ProP. Afterwards, all cDNA fragments were aligned by translatorX with default settings by muscle and the resulting alignment was cleaned by Gblocks with the default settings from the translatorX website. (TIFF) [file pone.0043036.s001.tiff]

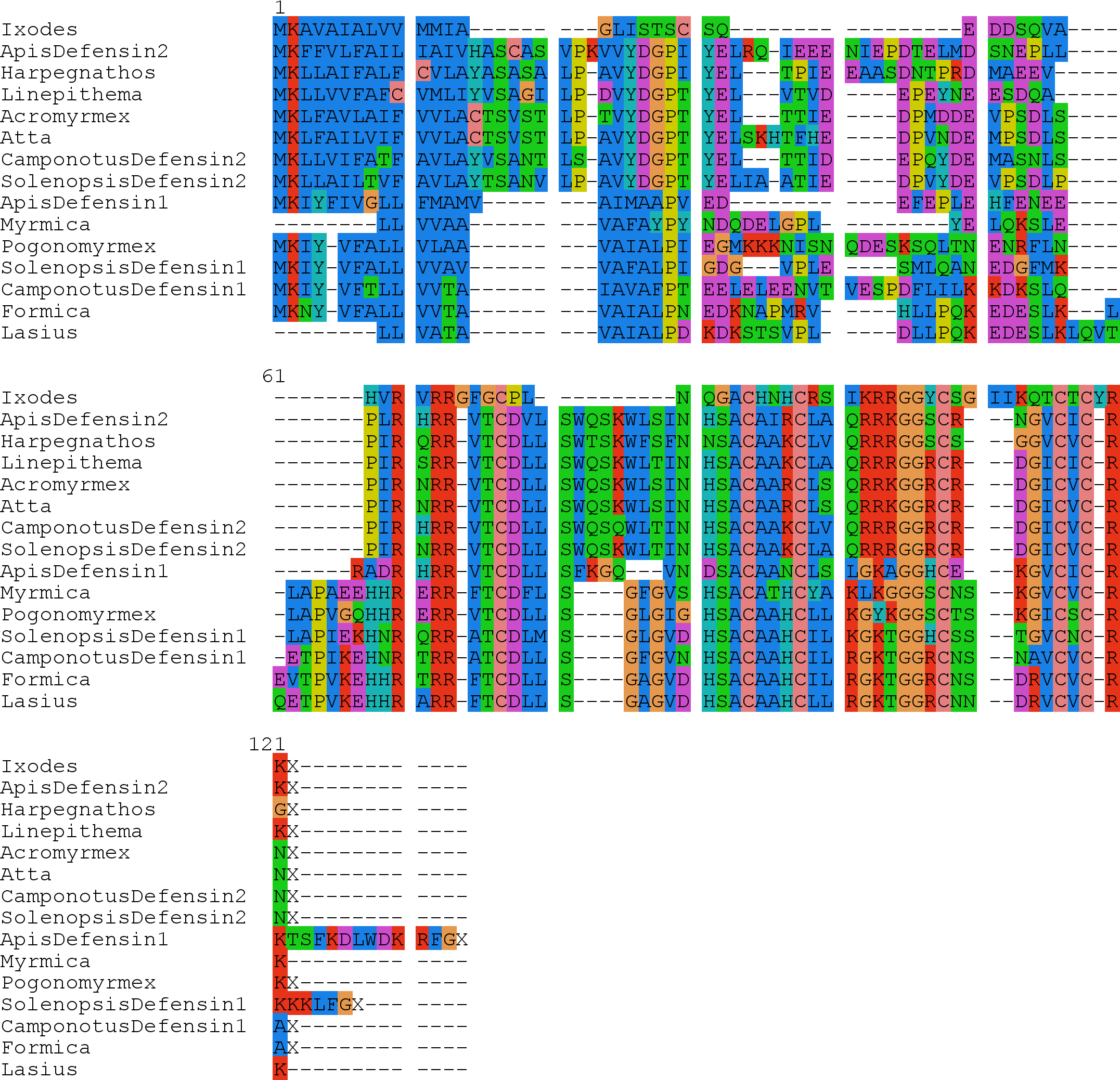

Supplement: Figure S2 — Alignment of defensin peptides from different ant species. The predicted peptide sequences for the seven ant species, the defensin-1 and defensin-2 of Apis mellifera (NM_001011616.2, NM_001011638.1), and the defensin of Ixodes scapularis (XP_002436104.1) were aligned by muscle with default settings. (TIFF) [file pone.0043036.s002.tiff]
