# Supplementary material for: Molecular Characterization of Antimicrobial Peptide Genes of the Carpenter Ant Camponotus floridanus
Source: PLoS One. 2012 Aug 9;7(8):e43036. doi: 10.1371/journal.pone.0043036 (PMC3415428; doi:10.1371/journal.pone.0043036)
Supplement: Table S1 — Primers used for characterization of C. floridanus hymenoptaecin and defensins. (PDF) [file pone.0043036.s003.pdf]

Supplementary Table S1: Primers used for characterization of *C. floridanus* hymenoptaecin and defensins.

Gene name	Primer name	Sequence 5´-3´	Application
hymenoptaecin (*hym*) (Acc. No.: HQ315784)	Cfl_hym_GSP1	GGCTGTGTGCCCTGGAAACTTGTCGAACCC	5´RACE
	Cfl_hym_GSP2	GACCCGATGCGCAAAGGAGGAGCTT	3´RACE
	Cfl_hym_flsF	GAAGTAGCTTCACAGTAGAAACGAAAA	Full length sequence
	Cfl_hym_flsR	TAATATTTTGTGAAACAGCCTCAAA	
	Cfl_hym_seqF1	AGCTTCCAGAGCCCGATGC	Sequencing (Seq.) primer
	Cfl_hym_seqR1	GCCGAAGTTCCTTTCAGC	
	Cfl_hym_introF1	CGCTTTCGCAAATGACTTCT	
	Cfl_hym_5´F	TGGCCTTATTGTGCGCTATC	5´-probe
	Cfl_hym_5´R	CGGTTGTGCGACCATTGTTA	
	Cfl_hym_repF	GGCATCCAGGCTGAAAGAAG	Repeat-probe
	Cfl_hym_repR	TCCTCCGTAGACATCCGCTG	
	Cfl_hymNdeI_F	CGCATATGCAAGGAACTTCACTAAGCT	cloning into pET-15b
	Cfl_hymBamHI_R	TAGGATCCTTAGAAGCGGTAGCCAGCACTTA	
defensin-1(*def-1*) (Acc. No.: JN989495)	Cfl_def-1_GSP1	TGCAGTGAGCTGCGCAAGCGCTATGAT	5´RACE
	Cfl_def-1_GSP2	TCATAGCGCTTGCGCAGCTCACTGCAT	3´RACE
	Cfl_def-1_flsF	ATTCTACTGCAAGATTTGAAAGACG	Full length sequence
	Cfl_def-1_flsR	AATTTCTGGTACATATACATTTGTTGA	
defensin-2 (def-2) (Acc. No.: JQ693412)	Cfl_def-2_GSP1	GACGACGCTGCACCAGGCATTTAGC	5´RACE
	Cfl_def-2_GSP2	CACCATCGACGAGCCGCAATACGAC	3´RACE
	Cfl_def-2_flsF	TCATTTCAGGGGTATTCGAGTG	Full length sequence
	Cfl_def-2_flsR	GCGATTGAGAAATTAAACTAACTGG	
	Cfl_def-2_F788	TTTCACTGTGGCAAACACACA	Seq. primer
